# Diversity of metabolite accumulation patterns in inner and outer seed coats of pomegranate: exploring their relationship with genetic mechanisms of seed coat development

**DOI:** 10.1038/s41438-019-0233-4

**Published:** 2020-01-07

**Authors:** Gaihua Qin, Chunyan Liu, Jiyu Li, Yongjie Qi, Zhenghui Gao, Xiaoling Zhang, Xingkai Yi, Haifa Pan, Ray Ming, Yiliu Xu

**Affiliations:** 1 Key Laboratory of Genetic Improvement and Ecophysiology of Horticultural Crops, Anhui Province, Horticultural Research Institute, Anhui Academy of Agricultural Sciences, Hefei, 230001 China; 2 Key Laboratory of Fruit Quality and Development Biology, Anhui Academy of Agricultural Sciences, Hefei, 230001 China; 3 FAFU and UIUC-SIB Joint Center for Genomics and Biotechnology, Fujian Agriculture and Forestry University, Fuzhou, 350002 China; 4 Department of Plant Biology, University of Illinois at Urbana-Champaign, Urbana, IL 61822 USA

**Keywords:** Developmental biology, Metabolism

## Abstract

The expanded outer seed coat and the rigid inner seed coat of pomegranate seeds, both affect the sensory qualities of the fruit and its acceptability to consumers. Pomegranate seeds are also an appealing model for the study of seed coat differentiation and development. We conducted nontarget metabolic profiling to detect metabolites that contribute to the morphological differentiation of the seed coats along with transcriptomic profiling to unravel the genetic mechanisms underlying this process. Comparisons of metabolites in the lignin biosynthetic pathway accumulating in seed coat layers at different developmental stages revealed that monolignols, including coniferyl alcohol and sinapyl alcohol, greatly accumulated in inner seed coats and monolignol glucosides greatly accumulated in outer seed coats. Strong expression of genes involved in monolignol biosynthesis and transport might explain the spatial patterns of biosynthesis and accumulation of these metabolites. Hemicellulose constituents and flavonoids in particular accumulated in the inner seed coat, and candidate genes that might be involved in their accumulation were also identified. Genes encoding transcription factors regulating monolignol, cellulose, and hemicellulose metabolism were chosen by coexpression analysis. These results provide insights into metabolic factors influencing seed coat differentiation and a reference for studying seed coat developmental biology and pomegranate genetic improvement.

## Introduction

Pomegranate (*Punica granatum* L.) is an ancient and beloved fruit known for its nutritional, medicinal, and ornamental importance. It has been cultivated since ancient times and is now planted in Mediterranean climates around the world, including mainly Tunisia, Turkey, Spain, Egypt, Morocco, the USA, China, India, Argentina, Israel, and South Africa^1^. The fleshy edible outer seed coats and hard or soft inner seed coat textures of various pomegranate cultivars affect the edibility of the fruit and the preferences of consumers for particular varieties. The edibility of pomegranates depends largely on the size of the outer seed coat. The texture of the inner seed coat greatly affects acceptability to consumers. The preference of most consumers for soft-seeded pomegranates has accelerated the breeding of soft-seeded pomegranate cultivars and promoted mechanistic studies of the development of soft inner seed coats^2–5^. However, a complete overview of seed coat development in pomegranate has not yet been obtained.

Analyses of the anatomical structure of pomegranate seed coats revealed two cell layers in the outer seed coat and three cell layers in the inner seed coat^6^, concordant with many other angiosperm species, including Arabidopsis^7^. The development of the seed coat is important for the establishment of a viable seed. For example, the absence of the endothelial integument layer or other seed coat defects can result in seed abortion^8^, which reflects the essential nature of seed coat differentiation during seed development.

The differentiation of seed coats begins with fertilization^9^, followed by cell expansion and growth, but not cell proliferation. The distinguishing morphological characteristics of inner and outer seed coats may be the consequences of the differential accumulation of diverse cellular metabolites. In Arabidopsis, the innermost layer of the inner integument synthesizes procyanidin (PA) flavonoids, while the subepidermal cells of the outer integument form a thickened cell wall known as the palisade^10^. The fleshy, edible outer seed coat of pomegranate seeds is rich in nutrients, such as sugars, organic acids, and anthocyanins^11^, while the cork layer and thick cell wall layer in the inner seed coat contain more lignins, cellulose, and hemicellulose^12,13^. Cellular components such as proteins, nucleic acids, signaling molecules, and organelles are also unevenly distributed between the inner and outer seed coats, which might modulate cell shape, structure, and function^14^. In this sense, metabolic profiling of the inner and outer seed coats of pomegranate will be of great significance for understanding the development of the seed coat in plants in general and the formation of seed coat texture in pomegranate in particular.

The compressed inner seed coat and expanded and fleshy outer seed coat of pomegranate differ significantly from those of many other plants, such as Arabidopsis and soybean^15^. The morphological characteristics of the pomegranate seed coat make it easy to isolate and collect the inner and outer seed coats from whole seeds, so it is an appealing subject for studying the development of seed coats.

In this study, nontargeted profiling of the metabolism of the inner and outer seed coats of two typical hard-seeded and soft-seeded pomegranate cultivars, *P. granatum* “Dabenzi” and *P. granatum* “Tunisia”, respectively, was performed at different developmental stages to study the accumulation of metabolites in these tissues and to identify metabolites that could contribute to seed coat development. The transcriptomes of corresponding tissues were analyzed to study the genetic control of the differential accumulation of these metabolites, as it relates to seed coat development. These results will provide insights into seed coat development in general, especially the development of inner seed coat hardness, and will be useful for identifying target genes to manipulate for breeding soft-seeded pomegranates that are preferred by consumers.

## Results

### Metabolite profiling of pomegranate seed coats

We performed untargeted metabolite analysis to determine global metabolic profiles in pomegranate seed coats and detected a total of 535 metabolites in inner and outer seed coats of the hard-seeded cultivar “Dabenzi” and soft-seeded cultivar “Tunisia” at three representative developmental stages of seed hardness formation. Our subsequent ‘omics analyses characterized different tissues (inner and outer seed coats) and genotypes (soft- and hard-seeded cultivars) at different developmental stages to reveal how the inner and outer seed coats differentiate and why inner seed coat development differs between soft- and hard-seeded pomegranate cultivars.

**Fig. 1:**
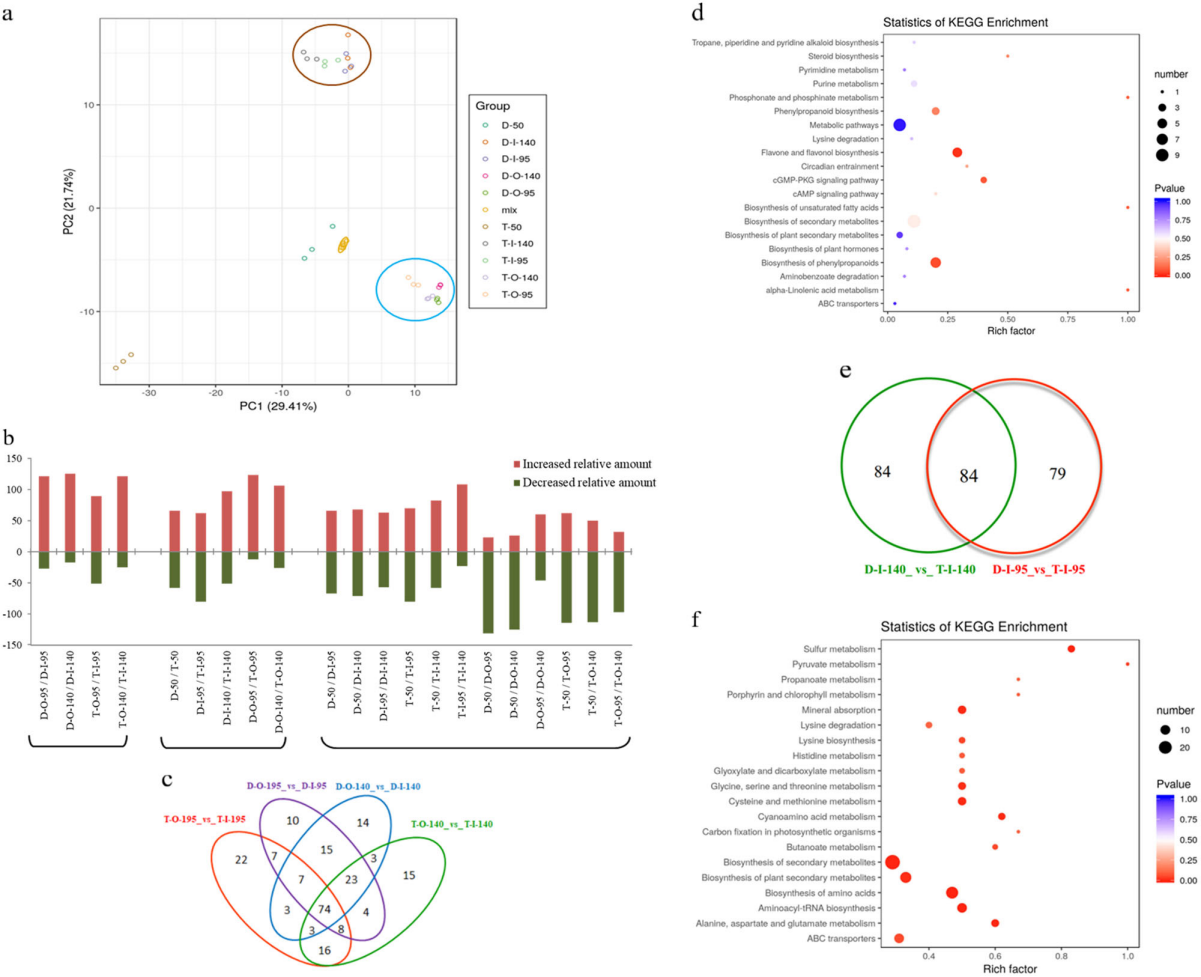
Principal component analysis (PCA) of metabolites and analysis of metabolites with differences in relative abundance between inner and outer seed coats at different developmental stages. **a** PCA of metabolites. **b** The numbers of metabolites with differences in relative abundance between inner and outer seed coats at different developmental stages. Red and green columns represent the numbers of metabolites with increases or decreases in relative abundance, respectively. The first letters in the designations on the *x*-axis represent abbreviations of the cultivar names: D for *P. granatum* “Dabenzi” and T for *P. granatum* “Tunisia”. The second letters in the designations represent tissues: “O” stands for the outer seed coat, and “I” stands for the inner seed coat. The subsequent number represents the number of days after flowering (DAF). “/” indicates a comparison of two samples. **c** Venn diagram of metabolites that differ in relative abundance between inner and outer seed coats of pomegranate. **d** KEGG enrichment analysis of metabolites that differed in relative abundance between inner and outer seed coats of pomegranate. **e** Venn diagram of metabolites that differed in relative abundance in inner seed coats between the pomegranate cultivars. **f** KEGG enrichment analysis of metabolites that differed in relative abundance in inner seed coats between the pomegranate cultivars.

First, we plotted metabolites with significantly differential abundance from all samples to obtain an overview of metabolic changes. Principal component analysis (PCA) of the metabolites showed that ~51% of the variance in metabolites between samples was explained by principal components 1 and 2. The high similarity among the three replicates indicated that the analysis was stable and repeatable. PCA revealed clear clustering of metabolic profiles according to tissue, but not according to genotype or fruit developmental stage (Fig. 1a). The distinct metabolic profiles between inner and outer seed coats indicated that distinct developmental differentiation occurred between the inner and outer seed coats.

**Table 1:** Sugars and sugar alcohols in seed coats of pomegranate.

Compounds	Fold change in relative amount between samples						
	D-50/ T-50	D-I-95/ T-I-95	D-I-140/ T-I-140	D-O-95/ D-I-95	D-O-140/D-I-140	T-O-95/ T-I-95	T-O-140/T-I-140
D-sorbitol	1.32	0.47	0.62	0.05	0.07	0.11	0.04
Pantothenol	0.23	0.95	1.06	0.79	0.95	217.86	1.46
D-mannitol	4.87	2.32	5.41	0.61	0.38	0.98	0.47
1,5-anhydro-D-glucitol	2.51	0.82	1.09	0.17	0.18	0.22	0.13
D(−)-threose	1.09	0.87	2.04	0.84	0.80	80.25	1.86
D( + )-melezitose O-rhamnoside	0.30	0.84	2.06	0.01	0.03	0.12	0.27
Glucarate O-phosphoric acid	1.52	0.75	0.37	31.02	33.13	16.95	20.67
Trehalose 6-phosphate	0.43	0.56	0.65	0.84	0.62	0.87	0.76
D( + )-melezitose	1.15	0.91	0.96	0.80	0.68	1.48	0.64
D-( + )-sucrose	1.48	1.18	1.03	0.29	0.26	0.57	0.30
D-( + )-glucono-1,5-lactone	1.08	2.39	2.06	0.71	0.41	3.55	1.22
D( + )-glucose	0.63	1.09	0.76	1.17	1.40	3.64	1.34
DL-arabinose	0.45	1.38	1.54	0.65	0.31	2.25	0.50
L-frucose	1.13	0.53	0.83	0.12	0.08	0.20	0.07
N-acetyl-D-glucosamine	0.46	0.74	0.72	3.09	1.63	1.73	1.13
D-glucose 6-phosphate	0.56	0.28	0.59	0.53	0.33	0.43	0.30
D-sedoheptuiose 7-phosphate	–	0.84	–	26.92	30.79	16.25	–
D-fructose 6-phosphate	0.64	0.33	0.64	0.43	0.30	0.43	0.29
Gluconic acid	3.28	4.35	10.77	0.22	0.06	1.62	1.15
D-glucuronic acid	2.40	2.04	3.08	2.09	0.79	3.73	3.63
D-xylonic acid	1.77	7.73	6.22	0.06	0.02	2.72	0.52

The metabolite was not detected in one or all of samples compared. The first letters in the sample designations represent abbreviations of the cultivar names: D for *P. granatum* “Dabenzi” and T for *P. granatum* “Tunisia”. The second letters represent tissues: “O” stands for the outer seed coat and “I” stands for the inner seed coat. The numbers following represent the number of days after flowering (DAF). Fold changes of metabolite relative amounts between samples were Log2 transformed

We then counted the number of metabolites with changed profiles in inner and outer seed coats of the two cultivars at two developmental stages and found that more metabolites had increased in quantity in outer seed coats than in inner seed coats. Comparison of metabolites with differential relative abundance between “Dabenzi” and “Tunisia” showed that more metabolites had increased in relative abundance in the inner seed coats of “Dabenzi” than in those of “Tunisia” at 140 DAF and that more metabolites had increased in relative abundance in the outer seed coats of “Dabenzi” than in those of “Tunisia” during all developmental stages analyzed (Fig. 1b).

Finally, we observed trends of changes in relative metabolite abundance during fruit development and found that the numbers of metabolites that had increased or decreased in relative abundance were similar for inner seed coats of “Dabenzi” and “Tunisia” during early development, while more metabolites had increased than decreased in relative abundance during later development (Fig. 1b). These trends indicated large increases in relative metabolite abundance in the inner seed coats later in development. For outer seed coats, more metabolites increased in relative abundance in “Dabenzi” later in development than early in development, although this trend was not observed for “Tunisia”. Therefore, the outer seed coats of “Dabenzi” accumulated more metabolites later in development than did the outer seed coats of “Tunisia”.

To gain insights into the possible contributions of these changed metabolites to tissue differentiation in seed coats, we visualized the metabolites that changed in common between the inner and outer seed coats in a Venn diagram (Fig. 1c). A total of 74 metabolites changed in common between the inner and outer seed coats of the cultivars “Dabenzi” and “Tunisia”. We assigned these metabolites to KEGG pathways and conducted an enrichment analysis. Notably, the pathways for phenylpropanoid biosynthesis and flavone and flavonal biosynthesis changed most significantly between the inner and outer seed coats in these two pomegranate cultivars (Fig. 1d).

To identify metabolites that might contribute to the development of inner seed coat texture, we focused on the metabolites in inner seed coats of the two cultivars and found that the abundances of 84 inner seed coat metabolites changed in abundance in both “Dabenzi” and “Tunisia” at 95 DAF and 140 DAF (Fig. 1e). We assigned these metabolites to KEGG pathways and found that the relative abundances of metabolites in pathways such as secondary metabolite and amino acid biosynthesis and metabolites transported by ATP-binding cassette (ABC) transporters differed significantly between the cultivars with different inner seed coat textures (Fig. 1f).

Phenolic compounds derived mainly from phenylpropanoids are common secondary metabolites in plants, and many phenolic compounds have been identified in seed coats. Epicatechin, cyanidin 3-O-glucoside, and delphinidin 3-O-glucoside are major phenolics in soybean seed coats, and epicatechin might be functionally related to coat-imposed seed hardness^16^. Phenolic compounds identified in the seed coats of *Acer truncatum* include gentisic acid, naringenin, epicatechin, procyanidin tetramers, and procyanidin pentamers^17^. Fischer et al. identified 48 phenolic compounds, including anthocyanins, ellagitannins, and other compounds that come from outer seed coats, in pomegranate juice^18^. In this study, we identified 23 flavones, 33 flavonols, 16 flavanones, 5 proanthocyanidins, 11 anthocyanidins, 11 catechin derivatives, and 6 isoflavones in pomegranate seed coats, and the total proportion of phenolic compounds ranged from 6% to 24% (Supplementary Fig. S1). Among these phenolic compounds, proanthocyanidins, anthocyanidins, flavanones, coumarins, and hydroxycinnamoyl derivatives were predominant.

### Differential accumulation of monolignols in inner and outer pomegranate seed coats and correlated expression of some genes related to monolignol biosynthesis

In pomegranate, the fleshy outer seed coat forms the edible pulp of the seed, while the inner seed coat becomes hardened due to thickened cell walls. Plant cell walls are composed of numerous polymers, including the polysaccharides cellulose and hemicellulose and the polyphenol lignin. Lignin is the major phenolic polymer in plant secondary cell walls (after cellulose) that fills the spaces in the cell wall between cellulose and hemicellulose polymers and confers rigidity to cell walls. Lignin is composed of the primary monolignols p-coumaryl alcohol, coniferyl alcohol, and sinapyl alcohol^19,20^, which are formed predominantly by oxidative polymerization of the three major monolignols to generate the hydroxyphenyl (H), guaiacyl (G), and syringyl (S) lignin subunits. In this study, relatively large amounts of coniferyl and sinapyl alcohols were detected in the inner and outer seed coats of pomegranate, while little p-coumaryl alcohol was detected in the inner seed coat of pomegranate. Thus, S lignin and G lignin appear to be the dominant lignin subunits in pomegranate seed coats.

**Fig. 2:**
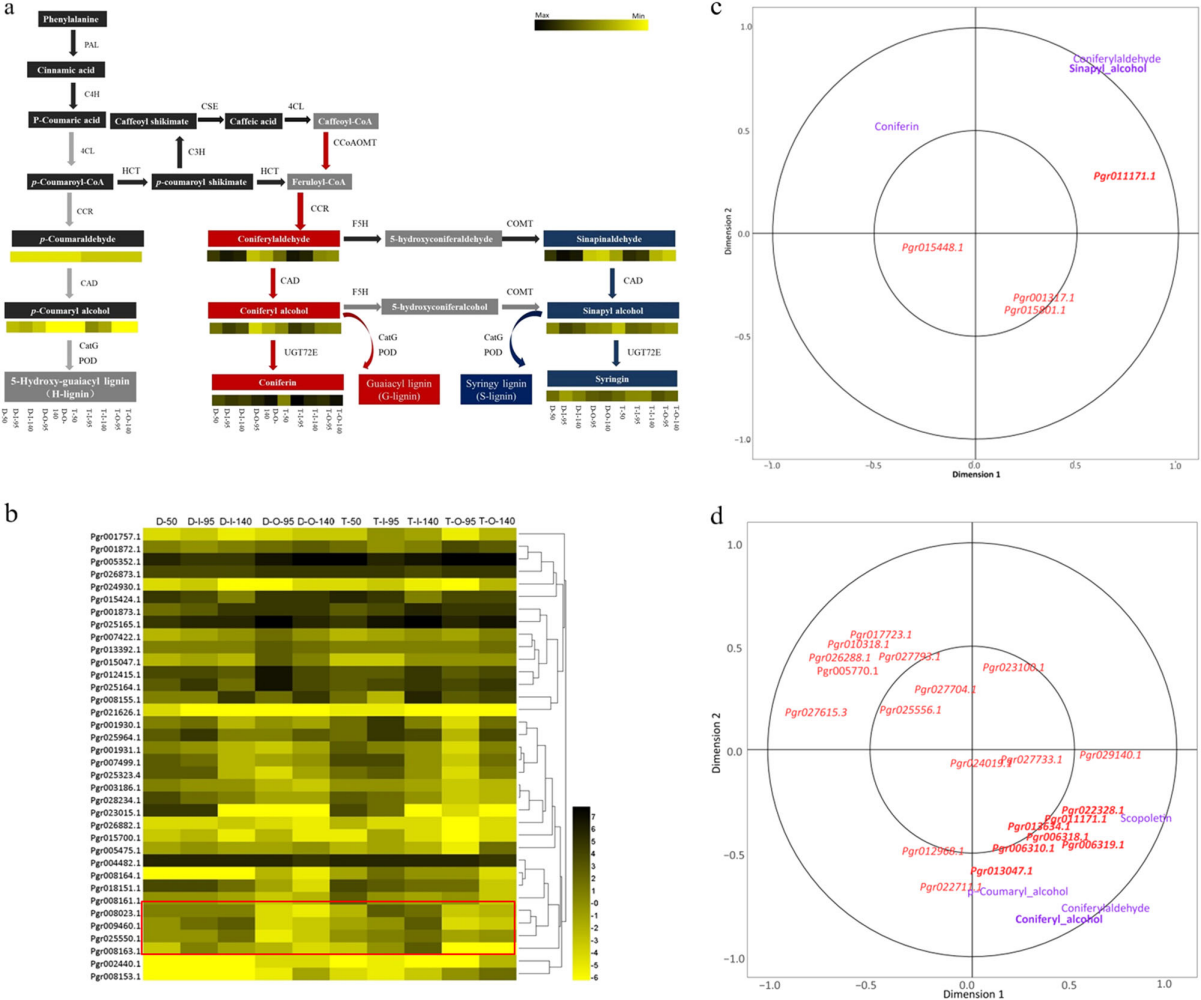
Schemas of monolignol biosynthesis and expression profiling of ABC transporters in pomegranate seed coats and canonical correlation analysis (CCA) of metabolites and genes involved in monolignol biosynthesis. **a** Schema of lignin biosynthesis in pomegranate seed coats. The predominant pathway for monolignol biosynthesis in xylem cells is outlined in black, while the minor pathway^43^ is outlined in gray. Compounds in the black boxes are components detected in this study, and those in gray boxes were predicted in a previous study of lignin biosynthesis^44^, but not detected in this study. The relative abundances of the compounds were log2 transformed. Bars on the right represent the relative abundances of metabolites; color shading from yellow to black indicates abundances from low to high. PAL L-phenylalanine ammonia-lyase, C4H cinnamate 4-hydroxylase, 4CL 4-coumarate: CoA ligase, CCR cinnamoyl-CoA reductase, CAD cinnamyl alcohol dehydrogenase, HCT hydroxycinnamoyl CoA: shikimate/quinate hydroxycinnamoyl transferase, C3′H 4-coumaroyl shikimate 3-hydroxylase, CCoAOMT caffeoyl-CoA 3-O-methyltransferase, COMT caffeic acid 3-O-methyltransferase, CSE caffeoyl shikimate esterase, F5H ferulate/coniferaldehyde 5-hydroxylase, UGT72E coniferyl alcohol glucosyltransferase, POD peroxidase, KatG catalase-peroxidase. **b** Expression profiling of ABC transporters in the seed coats of pomegranate. The first letters in the designations on the *x*-axis represent abbreviations of the cultivar names: D for *P. granatum* “Dabenzi” and T for *P. granatum* “Tunisia”. The second letters in the designations represent tissues: “O” stands for the outer seed coat, and “I” stands for the inner seed coat. The subsequent number represents the number of days after flowering (DAF). **c** CCA of metabolites and genes involved in sinapyl alcohol biosynthesis. **d** CCA of metabolites and genes involved in coniferyl alcohol biosynthesis. Metabolites are shown in red, and genes IDs are shown in purple. Farther distances in the same direction from the origin indicate stronger correlations.

To understand lignin formation and accumulation in pomegranate seed coats, we studied the accumulation of monolignols and their upstream phenylpropanoid metabolites in both the inner and outer seed coats of both cultivars and compared the accumulation of these metabolites in the inner seed coats between “Dabenzi” and “Tunisia”, which differ in seed hardness. We found that coniferyl alcohol and sinapyl alcohol, as their precursors coniferyl aldehyde and sinapyl aldehyde, accumulated considerably in inner seed coats at the later stage of fruit development and thus might be responsible for the greater lignification of inner seed coats. Although these metabolites exhibited similar accumulation patterns in the inner seed coats of cultivars with different seed hardnesses, the inner seed coats of “Dabenzi” accumulated more sinapyl alcohol than did the inner seed coats of “Tunisia” (Fig. 2a). Thus, sinapyl alcohol was the main monolignol that differed in abundance between the hard inner seed coats of “Dabenzi” pomegranate seeds and the soft inner seed coats of “Tunisia” pomegranate seeds. At the same time, the monolignol glucosides coniferin and syringin showed accumulation patterns nearly the opposite of those of coniferyl alcohol and sinapyl alcohol. The inner seed coat accumulated less coniferin and syringin than did the outer seed coats, and the relative accumulation of these compounds in “Dabenzi” was lower than that in “Tunisia”. A model for metabolic fluxes in pomegranate seeds might predict that larger amounts of coniferyl alcohol and sinapyl alcohol in the inner seed coats of hard-seeded cultivars would be transported to the cell wall and polymerized into lignin, while the larger amounts of coniferyl alcohol and sinapyl alcohol in the inner seed coats of soft-seeded “Dabenzi” and the outer seed coats of both cultivars would be glycosylated into monolignol glucosides and then stored.

To learn more about monolignol fluxes, we studied the correlations between monolignol formation and the expression of genes encoding proteins involved in their transport. ABC transporters are involved in the transmembrane transport of monolignols^21^, among other compounds. We identified 47 genes encoding ABC transporters in the pomegranate genome and conducted coexpression analysis of these genes and genes encoding proteins involved in monolignol biosynthesis and determined that 36 putative ABC genes were coexpressed with genes potentially involved in monolignol biosynthesis (Supplementary Fig. S2). We then analyzed the expression of ABC genes in the seed coats of the two cultivars and found that *Pgr008023*, *Pgr008163*, *Pgr009460*, and *Pgr025550*, which encode ABC transporters and are homologous to *AtABCG9*, *AtABCG11*, *AtABCG21*, and *AtABCG24*, respectively, which encode proteins and are predicted to localize to the plasma membrane (http://suba.live/), were specifically expressed in the inner seed coats (Fig. 2b; Supplementary Fig. S3). The accumulation of these transcripts in inner seed coats and the amounts of the putative ABC transporters that they encode (Fig. 2d, f) might contribute to the accumulation of the kinds of metabolites transported by ABC transporters. Because monolignol aglycones can be translocated by ABC transporters, which are located on the plasma membrane^22^, these data are an important part of our model explaining lignin formation in inner seed coats, especially in the hard-seeded cultivar “Dabenzi”.

Lignification is a complex biological process that involves many cellular and metabolic processes controlled by networks of genes and metabolites. Metabolite fluxes result from the spatiotemporal coordination of several metabolic processes involving many genes. Systems biology studies previously integrated several types of ‘omics data across developmental stages or plant organs to elucidate the networks involved in many processes in plants^23^. In this study, we identified homologous genes in the entire pomegranate genome encoding 14 enzymes involved in lignin biosynthesis, and measured their transcript profiles relative to those of metabolites in inner and outer seed coats of “Dabenzi” and “Tunisia” pomegranate seeds at different developmental stages. Regularized canonical correlation (RCC) analysis of the sinapyl alcohol content in these tissues and the expression of related genes showed that the expression of *Pgr011171.1*, which encodes peroxidase, was highly correlated with the amount of sinapyl alcohol in the inner seed coats of “Dabenzi” at DAF 50 (Fig. 2c). Therefore, *Pgr011171.1* is a candidate gene for regulating the biosynthesis of sinapyl alcohol in the inner seed coat of “Dabenzi” during early development. Similarly, strong correlations were observed in pomegranate seed coats between the coniferyl alcohol content and the expression of several genes, including *Pgr013634.1*, which encodes ferulate-5-hydroxylase (F5H); *Pgr022328.1*, *Pgr006310.1*, *Pgr006318.1*, and *Pgr006319.1*, which encode cinnamyl alcohol dehydrogenase (CAD); and *Pgr011171.1*, which encodes peroxidase (POD) (Fig. 2d). These observations imply that the more extensive accumulation of coniferyl alcohol in inner seed coats than in outer seed coats might contribute to the greater expression of *Pgr022328.1*, *Pgr013634.1*, *Pgr006318.1*, *Pgr006319.1*, and *Pgr011171.1*.

### Differential accumulation of cellulose and hemicellulose in inner and outer pomegranate seed coats and correlated expression of some genes related to cellulose and hemicellulose biosynthesis

Hemicellulose is a polysaccharide consisting of several different sugar monomers, while cellulose is a polysaccharide consisting of only anhydrous glucose. Both unmodified sugars and their acidified forms can be found in hemicellulose; for instance, glucuronic acid and galacturonic acid can be found in hemicellulose^24^. We detected 21 sugars, saccharic acid and sugar alcohols in pomegranate seed coats (Table 1). Among these compounds, gluconic acid and D-xylonic acid were the predominant saccharic acid monomers in the inner seed coats.

To understand the differential accumulation of hemicelluloses in the coats of seeds that differ in hardness, we compared hemicellulosic metabolites in the inner and outer seed coats of the cultivars “Dabenzi” and “Tunisia”. In contrast to outer seed coats, the inner seed coats accumulated substantial amounts of D( + )-melezitose O-rhamnoside, D-( + )-sucrose, L-fructose, D-fructose 6-phosphate, D-glucose 6-phosphate, 1,5-anhydro-D-glucitol, and D-sorbitol. D-( + )-glucono-1,5-lactone, gluconic acid, and D-xylonic acid accumulated differentially between inner and outer seed coats only in the hard-seeded cultivar “Dabenzi”.

Comparisons of hemicellulosic metabolites in the inner seed coats between “Dabenzi” and “Tunisia” seeds showed that gluconic acid, D-( + )-glucono-1,5-lactone, D-glucuronic acid, D-xylonic acid, and D-mannitol accumulated in greater relative abundances in the hard-seeded cultivar “Dabenzi” than in the soft-seeded cultivar “Tunisia”, while D-fructose 6-phosphate, D-glucose 6-phosphate, and trehalose 6-phosphate accumulated less in the hard-seeded cultivar than in the soft-seeded cultivar. Our comparison indicated that gluconic acid, D-( + )-glucono-1,5-lactone, and D-xylonic acid were the predominant constituents of the hemicellulose in the inner seed coat of hard-seeded “Dabenzi”, but not in its outer seed coat and in contrast to the pattern observed for the soft-seeded cultivar “Tunisia”.

We identified genes encoding 17 enzymatic steps in the biosynthesis of cellulose monomers and hemicelluloses (heteroxylans) and a gene encoding a carbohydrate-active enzyme (CAZyme), which synthesizes, modifies, and degrades cellulose and hemicelluloses^25^. Most of these genes belong to multigene families, but only a few exhibited high transcript accumulation during seed coat development. Expression profiling revealed that most of these genes were more highly expressed in inner seed coats than in outer seed coats, and that “Dabenzi” and “Tunisia” exhibited similar patterns of expression for these genes (Supplementary Fig. S4).

Among the enzymes encoded by these genes, cellulose synthase (CesA) usually acts as part of cellulose synthase complexes (CSCs) that include several catalytic subunits known as CesA proteins^26^. CesA 4, 7, and 8 are subunits that assemble into CSCs and biosynthesize cellulose in plant secondary cell walls^27^. We identified 13 *CesA* genes in the pomegranate genome that might encode CesA subunits. *Pgr007560*, *Pgr012627*, and *Pgr027371*, whose transcripts accumulated to high levels in inner seed coats, encode CesA 4, 7, and 8 (Supplementary Figs. S4, S5), which could contribute to cellulose biosynthesis in the inner pomegranate seed coat.

**Fig. 3:**
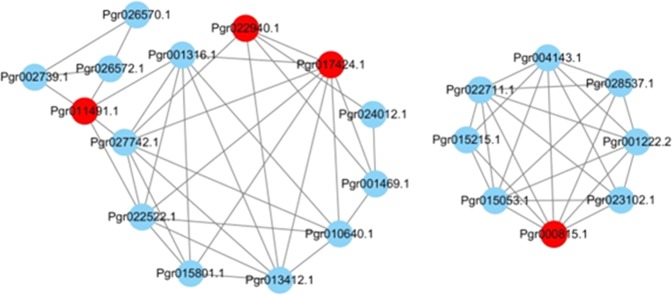
Coexpression of genes and transcription factors (TFs) involved in lignin, cellulose, and hemicellulose biosynthesis and accumulation. Only genes with correlation coefficients > 0.95 are shown. Red circles represent structural genes; blue circles represent TFs.

Transcription factors (TFs) including NAC, R2R3-MYB, AUX-IAA, and WRKY regulate the expression of genes involved in lignin^28^, cellulose, and hemicellulose biosynthesis^25^. Therefore, we analyzed the coexpression of these TFs and genes encoding enzymes that participate in lignin, cellulose, and hemicellulose biosynthesis in the inner and outer seed coats of hard-seeded “Dabenzi” and soft-seeded “Tunisia”. We found that *Pgr000815*, which encodes AUX-IAA, was coexpressed with genes in the PAL pathway. We also found that *Pgr011491*, which encodes AUX-IAA2; *Pgr022940*, which encodes MYB; and *Pgr017424*, which encodes NAC66, were coexpressed with genes involved in cellulose and hemicellulose biosynthesis (correlation indices > 95) (Fig. 3). Hence, we concluded that *Pgr000815* might be involved in the regulation of lignin biosynthesis and that *Pgr011491*, *Pgr022940*, and *Pgr017424* might be involved in the regulation of cellulose and hemicellulose biosynthesis in pomegranate seeds.

### Differential biosynthesis and accumulation of flavonoids in seed coats and correlated expression of some genes related to flavonoid biosynthesis

**Fig. 4:**
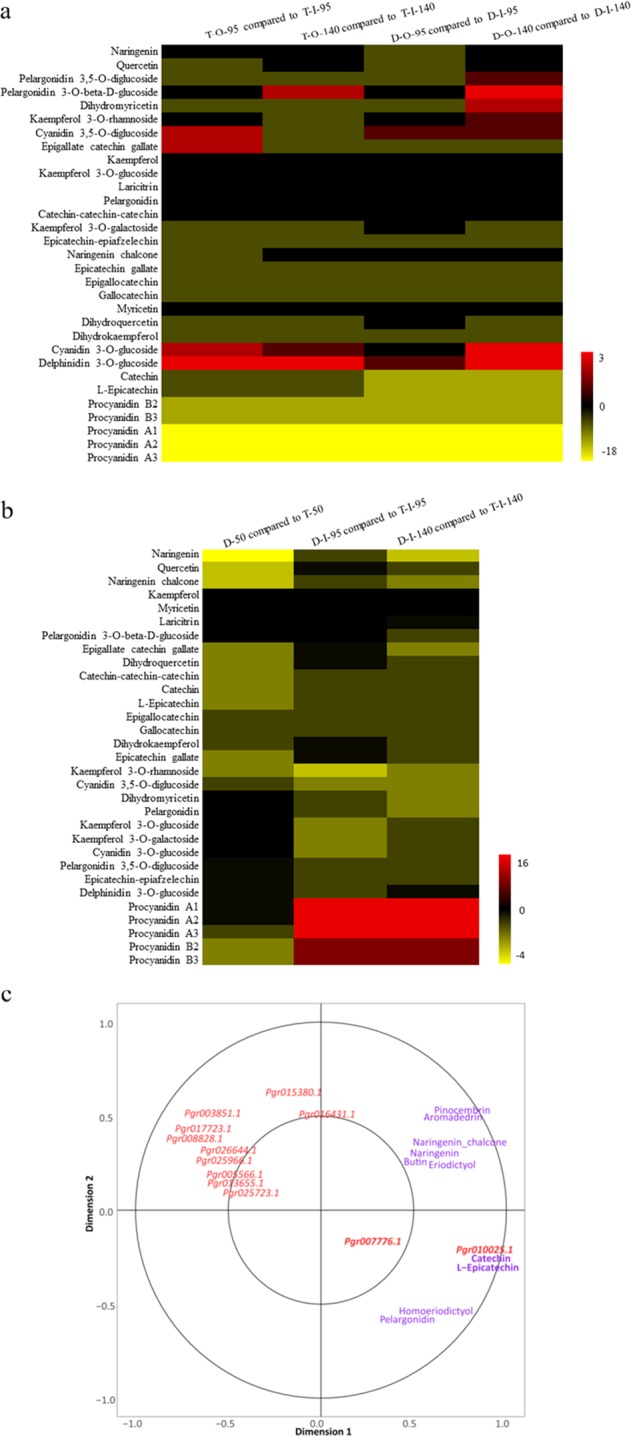
Comparative analysis of flavonoid compounds in the seed coats of pomegranate and CCA of metabolites and genes involved in flavonoid biosynthesis. **a** Comparative analysis of flavonoid compounds in the inner and outer seed coats of pomegranate. **b** Comparative analysis of flavonoid compounds in the inner seed coats of two pomegranate cultivars. The first letters in the designations on the *x*-axis represent abbreviations of the cultivar names: D for *P. granatum* “Dabenzi” and T for *P. granatum* “Tunisia”. The second letters in the designations represent tissues: “O” stands for the outer seed coat, and “I” stands for the inner seed coat. The subsequent number represents the number of days after flowering (DAF). Ratios of the relative metabolite contents were log2 transformed. Bars on the right indicate ratios from high to low. **c** CCA of metabolites and genes involved in flavonoid biosynthesis. Metabolites are shown in red, and genes IDs are shown in purple. Farther distances in the same direction from the origin indicate stronger correlations.

Flavonoids comprise a wide range of polyphenols derived from phenylpropanoids and are synthesized in a branched pathway that yields both colorless compounds (e.g., flavone and flavonols) and colored pigments (e.g., anthocyanins and proanthocyanidins)^29,30^. Proanthocyanidins (PAs), which are oligomers and polymers of monomeric flavonoids, are the end products of the flavonoid biosynthetic pathway. In this study, flavonoids including chalcone, flavones, flavonols, dihydroflavonol, anthocyanins, and PAs were detected in pomegranate seed coats. To reveal the flavonoid biosynthesis and accumulation patterns in the inner and outer seed coats of pomegranate seeds, we analyzed the flavonoids present in inner and outer seed coats and identified catechin and L-epicatechin as the main flavanols that accumulated to a relatively large degree in inner seed coats. Cyanidin 3-O-glucoside, delphinidin 3-O-glucoside, pelargonidin 3-O-beta-D-glucoside, cyanidin 3,5-O-diglucoside, and pelargonidin 3,5-diglucoside were the anthocyanins that accumulated to the greatest extent in the outer seed coats (Fig. 4a). Large amounts of PAs, also called condensed tannins, accumulated in the inner seed coats of pomegranate, as in Arabidopsis^31^. Among these, procyanidins A1, A2, and A3 were the primary PAs that accumulated in the inner seed coats of pomegranate. Comparison of the flavonoid contents of the inner seed coats between hard-seeded “Dabenzi” and soft-seeded “Tunisia” showed that the hard-seeded cultivar accumulated more PAs than did the soft-seeded cultivar (Fig. 4b).

Although the structural and regulatory genes controlling flavonoid biosynthesis have already been identified^32^, it is still not yet clear which genes control the synthesis of which class(es) of flavonoids and their accumulation in different tissues. Thus, we performed RCC analysis of the tissue contents of these compounds and the expression of genes in related biosynthetic pathways to identify candidate genes. We found that catechin and L-epicatechin contents were significantly correlated with transcript expression of *Pgr007776.1*, which encodes ANS (leucoanthocyanidin dioxygenase), and *Pgr010025.1*, which encodes DFR (bifunctional dihydroflavonol 4-reductase/flavanone 4-reductase) (Fig. 4c). The enzymes encoded by these genes are involved in the transition between dihydroflavonol and leucoanthocyanidin^33^, which would be critical for the synthesis of catechin and L-epicatechin in pomegranate seed coats.

### Expression profiles of genes related to integument development and auxin biosynthesis and function in seed coats

The degree of seed coat development determines pomegranate yield and, therefore, economic benefits for pomegranate planters because the size of the seed coat, especially the fleshy outer seed coat, determines the edibility of the fruit. In general, fruit growth occurs during an initial proliferative phase in which cell numbers increase while their size remains fairly constant, followed by a dramatic cell size increase. Seed coats begin to develop after the integuments differentiate into different cell layers. Many genes involved in integument development^7^ then continue to play roles in seed coat development and growth^5^. We surveyed the expression patterns of several of these kinds of genes and found that they were very similar between “Dabenzi” and “Tunisia” (Supplementary Fig. S6), which implied similar patterns of development of the seed coat in these two cultivars.

Differential patterns of expression of these genes between inner and outer seed coats could contribute to the distinct development of the seed coats. For example, DSO (ATP-binding cassette G11) is an ABCG-type transporter that exports cutin and waxes, the main components of the cuticle, across the plasma membrane to the cell wall and then to the extracellular matrix^34,35^. High expression of DSO in the inner seed coat might be responsible for the prominent cuticular layer of the inner seed coats in pomegranate.

Genes, especially those expressed later in development, orthologous to *TRANSPARENT TESTA GLABRA 1* (*TTG1*), which is involved in integument development through cell expansion^36^, were more highly expressed in the inner seed coats than in the outer seed coats. Genes orthologous to *TRANSPARENT TESTA GLABRA 2* (*TTG2*), another gene involved in integument development through cell expansion^36^, and *KANADI1* (KAN1), a gene that regulates auxin biosynthesis, transport, and signaling in Arabidopsis^37^, were both more highly expressed in the outer seed coats than in the inner seed coats of pomegranate, which might explain the vacuolation of cells in the outer seed coats.

Auxin has known roles in seed coat formation. For example, auxin can trigger seed coat formation without any other signals^9,33^. Similarly, seed coat development, which involves more cell elongation than cell proliferation, is triggered by auxins after fertilization^38^. In this study, we investigated the expression profiles of genes involved in auxin biosynthesis, transport, and signaling and found that they were very similar between “Dabenzi” and “Tunisia”. However, we found that genes involved in auxin biosynthesis and transport were more highly expressed in the inner seed coats than in the corresponding outer seed coats (Supplementary Fig. S7), suggesting that the formation of the fleshy outer seed coat might not be due to extraordinary developmental processes. The development of the inner and outer seed coats might result from a balance between the expression of genes encoding proteins involved in auxin biosynthesis, transport, and signaling that might regulate integument development through interaction as a protein complex^33^.

### Confirmation of the transcriptome data using qRT-PCR

**Fig. 5:**
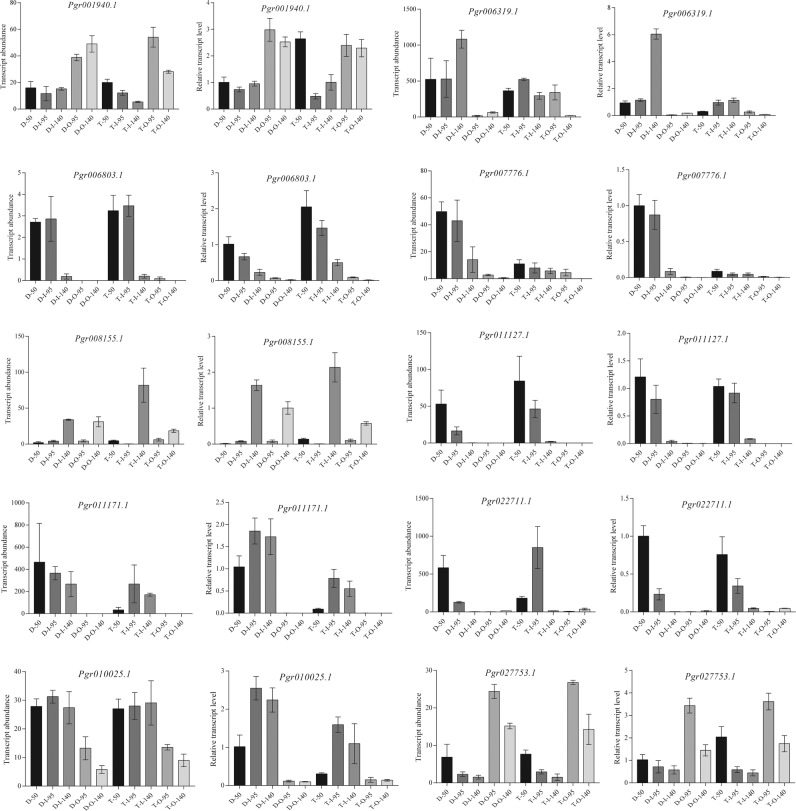
Validation of transcriptomic data using qRT-PCR. Transcript abundances detected by transcriptome sequencing (A) and expressed in FPKM are shown on the left. Relative expression detected by qRT-PCR (B) and expressed in 2^-ΔΔct^ is shown on the right. Data are the means of three replicates with three biological repeats. Error bars indicate SEs.

Ten genes were selected randomly from among the candidate genes for seed coat differentiation and development, and their expression was analyzed using qRT-PCR to validate the transcriptome data sets from RNA-Seq. The qRT-PCR results for the candidate genes were in close agreement with the corresponding relative transcript abundances from RNA-Seq in all cases (Fig. 5), validating the RNA-Seq results for the expression of these candidate genes.

## Discussion

The architecture of the seed coat reflects its evolutionary adaptation to different environments and reproductive strategies. Seed size depends on seed coat development, which influences plant survival and seed yield^39^. Therefore, seed coat development has attracted the attention of botanists and biologists.

As observed in most angiosperms, pomegranate has two seed coats that originate from the inner and outer integuments. However, the cell layers in inner and outer seed coats then follow distinct developmental programs after differentiation and show different patterns in terms of metabolites and morphological characteristics. The differential cell expansion and differences in molecular composition and metabolite accumulation result in a fleshy, edible outer seed coat and firm inner seed coat. The spatial expression patterns of TTG1 and DSO in inner seed coats and TTG2 and KAN1 in outer seed coats combined with differential metabolite accumulations might begin to explain the formation of these distinctive inner and outer seed coats. The flavonoids procyanidin A1, procyanidin A2, procyanidin A3, catechin, and L-epicatechin accumulate mainly in the inner seed coats of pomegranate. Gluconic acid, D-( + )-glucono-1,5-lactone, and D-xylonic acid are the predominant hemicellulose constituents of the inner seed coat. Finally, high relative accumulations of the main monolignols coniferyl alcohol and sinapyl alcohol were also found in inner seed coats. Understanding the diversity of metabolite accumulation patterns in different tissues should provide insights into the morphogenesis of the inner and outer seed coats and explain the mechanisms of development of pomegranate seed coat texture. The diverse pathways involved in seed coat development in pomegranate make this species a good model for studying seed biology. Thus, the metabolic and transcriptomic findings of this study provide vital insights into seed coat development in plants in general and in pomegranate in particular.

It is important to understand the synthesis, transport, and degradation of metabolites, as well as the molecular mechanisms underlying specialized metabolism, to explain metabolite accumulation. Forward and reverse genetics have been used alone or in combination to better understand the genetic variation that controls the metabolic diversity in plants^40^. In pomegranate, secondary metabolites including flavonoids, lignin, and other compounds derived from the phenylpropanoid pathway significantly differ between the inner and outer seed coats, and the lignin content of the inner seed coats of different pomegranate varieties correlates with their seed hardness^12^. In the past decade, no studies on the metabolism of developing seed coats have been conducted, while many studies on the development of seed hardness have focused on genes involved in lignin biosynthesis and differences in their expression detected via transcriptomic or proteomic analysis. Nevertheless, it is difficult to further narrow the large set of candidate genes to select target genes for future studies without more information. Combining genomic, transcriptomic, proteomic, and metabolomic data sets can facilitate the identification of candidate genes. Identifying candidate genes and TFs by using correlation analysis of transcripts and metabolites will shed light on lignin, cellulose, hemicellulose, and flavonoid biosynthesis, especially in regard to pomegranate seed coat development.

From the perspective of metabolic flux, monolignols synthesized in the cytoplasm have two possible destinies: storage in the cytoplasmic vacuoles or transport to the cytomembrane^41^, followed by oxygenation and polymerization into lignin^42^. Hence, the transport of monolignols is as important as their synthesis, which contributes to lignification, cell wall rigidity, and seed hardness. The identified candidate genes encoding candidate ABC transporters provided insights into the transmembrane transport of monolignols in pomegranate. Further analysis of the functions of these candidate genes should improve our understanding of the lignification of inner seed coats. ABC transporters encoded by *AtABCG9* and *AtABCG11* are required for vascular development in Arabidopsis, possibly as transporters of monolignol^42^. Might candidate genes in pomegranate homologous to *AtABCG9* and *AtABCG11* affect seed hardness by a similar mechanism? Further biochemical or biological studies of the functions of these candidate genes will be needed to understand monolignol transport and lignification of the cell wall and unravel the mechanism of seed hardness development.

## Materials and methods

### Plant materials

*P. granatum* “Dabenzi”, a hard-seeded pomegranate cultivar, and *P. granatum* “Tunisia”, a soft-seeded cultivar, were planted in an orchard (Hefei, 31°51'9.05“N, 117°06'34.33“E) in Anhui Province in China and grown under the same agronomic practices, including fertilization and irrigation. Five fruits from individual trees were harvested at random and pooled for each biological replicate to study seed coat development and morphological differentiation. Seeds from fruits were chosen at 50, 95, and 140 days after flowering (DAF) as representative examples of seed hardness in the hard-seeded pomegranate cultivar “Dabenzi”^5^ and at the corresponding developmental stages from fruits of the soft-seeded cultivar “Tunisia”.

Cleaned fresh fruits were dissected using a blade on a clean table, and seeds were stripped manually while wearing sterile gloves. The outer seed coats of fruits collected 95 DAF and 140 DAF were obtained by manually squeezing the whole fruit with sterile gauze, freezing the juice immediately in liquid nitrogen, and storing it at −80 °C until further use. The remaining inner seed coats were rubbed with sterile gauze to completely remove the outer seed coat, frozen in liquid nitrogen, and stored at −80 °C until further use. For seeds from fruits collected 50 DAF, the whole seed was used because it was difficult to visually distinguish the inner and outer seed coats. Samples from the same tree were considered one biological replicate. Three biological replicates were taken for RNA sequencing and metabolic profiling. In the present experiment, the inner (I) and outer (O) seed coats of “Dabenzi” (D) and “Tunisia” (T) pomegranates at 50, 95, and 140 DAF were abbreviated as follows: D-50, D-I-95, D-O-95, D-I-140, D-O-140, T-50, T-I-95, T-O-95, T-I-140, and T-O-140 (Supplementary Table S1).

### Untargeted metabolic profiling

The freeze-dried inner and outer seed coats were crushed with zirconium beads using a mixer mill (MM 400, Retsch, Germany) for 1.5 min at 30 Hz. Then, 100 mg of powdered tissue was dissolved in 1.0 mL of H_2_O/methanol (30:70 v/v) and blended. Extraction was carried out in the dark at 4 °C for 24 h, and then the homogenate was centrifuged at 10,000 × *g* for 10 min to remove the undissolved residue. The extracts were absorbed onto a CNWBOND Carbon-GCB SPE Cartridge (ANPEL, Shanghai, China) and filtered with an SCAA-104 membrane (0.22 µm, ANPEL, Shanghai, China) before being subjected to LC-MS analysis.

Metabolites were analyzed using a liquid chromatography–electrospray ionization-tandem mass spectrometry (LC–ESI–MS) (LC, Shim-pack UFLC Shimadzu CBM30A system; ESI, MS, Applied Biosystems 6500 QTRAP®) system equipped with a C18 column (1.8 µm, 2.1 mm × 100 mm, Waters ACQUITY UPLC HSS T3). Water and acetonitrile with 0.04% acetic acid (v/v) were used as mobile phases A and B, respectively. The column temperature was maintained at 40 °C, and the flow rate was 0.4 mL/min. The injection volume was 2 μL. Gradient elution was performed as follows: 0–11 min, 95 A:5B (v:v); 11–12 min, 5 A:95B (v:v); and 12.1–15 min, 95 A:5B (v:v). The effluent was alternatively connected to an ESI-triple quadrupole-linear ion trap (QTRAP)-MS. Linear ion trap (LIT) and triple quadrupole (QQQ) scans were acquired on an API 6500 QTRAP LC/MS/MS system equipped with an ESI turbo ion-spray interface operating in positive ion mode and controlled by Analyst 1.6 software (AB Sciex). The ESI conditions included turbo spray, 5500 V, and 550 °C. Ion source gas I (GSI), ion source gas II (GSII), and the curtain gas (CUR) were at 55, 60, and 25 psi, respectively, and the collision gas (CAD) pressure was set to high.

Instrument tuning and mass calibration were performed with 10 and 100 μmol/L polypropylene glycol solutions in QQQ and LIT modes, respectively. QQQ scans were acquired when the collision gas (nitrogen) pressure for MRM experiments was at 5 psi. For MRM transitions, declustering potential (DP) and collision energy (CE) were required each time.

Qualitative analysis of MS data was carried out by comparing the accurate precursor ion (Q1) values, product ion (Q3) values, retention times (RTs), and fragmentation patterns with those obtained by injecting standards under the same conditions, if the standards were available (Sigma-Aldrich, USA). If the standards were not available, analyses were conducted using the MWDB database (MetWare Biological Science and Technology Co., Ltd.) and the publicly available metabolite databases online at PLANTCYC (http://www.plantcyc.org/), the Kyoto Encyclopedia of Genes and Genomes (KEGG) (http://www.kegg.jp/), and an in-house SPL program (http://spldatabase.saskatoonlibrary.ca/).

Quantitative analysis was performed in MRM mode. The characteristic ions for each metabolite were screened through QQQ MS to obtain their signal strengths. Integration and correction of chromatographic peaks were performed using MultiQuant version 3.0.2 (AB SCIEX, Concord, Canada). The corresponding relative metabolite contents were represented as chromatographic peak area integrals. Screening and quantitative analysis for differential metabolites were conducted using metaX software (http://metax.genomics.cn/).

The criterion for significance (*P*-value) was set to 0.05, and the minimum fold change was set to 2.0. Counts of differential metabolites are shown as Venn diagrams. Enriched terms for differential metabolites were determined according to the KEGG database with *E*-values = < 1e-10 and were clustered using the clusterProfiler R package (version 3.6.0).

### RNA isolation and transcriptome sequencing

RNA-Seq was performed using RNA extracted from inner and outer pomegranate seed coats using TRIzol reagent (Qiagen, Beijing, China) and purified using an RNeasy Mini Elute Cleanup Kit (Qiagen, Beijing, China). Sequencing libraries were generated using the NEBNext Ultra^TM^ RNA Library Prep Kit for Illumina (New England Biolabs, USA) following the manufacturer’s instructions. Sequencing was carried out on an Illumina HiSeq 2000 platform to generate paired-end reads. Adaptor sequences and low-quality sequence reads (identity ≥ 95% and coverage length ≥ 100 bp) were removed, and clean reads were mapped to the reference “Dabenzi” pomegranate genome sequence (https://www.ncbi.nlm.nih.gov/nuccore/MTKT00000000.1) using HISAT2 release 2.1.0 (https://ccb.jhu.edu/software/hisat2/index.shtml). Putative gene functions were annotated using the following databases: Nr (NCBI nonredundant protein sequences; http://www.ncbi.nlm.nih.gov/RefSeq/), SWISS-PROT (http://www.UniProt.org/), KO (the KEGG Orthology database; http://www.genome.jp/kegg/ko.html), and GO (Gene Ontology; http://www.geneontology.org/).

### Gene identification and expression analysis

Orthologs of 11 enzymes involved in monolignol biosynthesis were identified by alignment to KEGG Orthology entries in the phenylalanine pathway (Ko00940) using blastx (*E*-value ≤ 1e-20 and identity ≥ 60%).

Genes encoding ABC transporters in the pomegranate genome were queried using the domains ABC2_membrane (PF01061.24) and ABC_transporter (PF00005.27) in HMMER software and then identified using phylogenetic trees for *PgrABC* and *AtABC* genes (Supplementary Fig. S3). TFs including NAC, MYB, AUX-IAA, and WRKY were identified in the same manner by querying separately with these domains from the putative proteins PF02365.15, PF00249, PF02309.16, and PF03106.15.

Homologs of genes encoding proteins likely involved in auxin biosynthesis, transport, and signaling were identified by aligning predicted proteins to the SWISS-PROT database (release-2015_04) using blastx with an identity ≥ 40% and a reference coverage ≥ 50%. Genes identified in this manner included homologs encoding proteins that perform the 18 enzymatic steps of cellulose and heteroxylan biosynthesis, as well as carbohydrate-active enzymes (CAZymes), and were the same as those described by Myburg et al.^25^. Homologous genes likely involved in integument development were also the same as those described by Qin et al.^5^.

Transcriptional abundances were estimated using the fragments per kilobase of exon per million mapped reads (FRKM) method, normalized by log2 FRKM transformation, and expressed as a heat map generated using heml 1.0 (http://hemi.biocuckoo.org/).

### RCC and coexpression analysis

We performed RCC to identify potential associations between metabolites and genes expressed in pomegranate. Canonical correlation analyses (CCAs) between metabolites and genes involved in monolignol biosynthesis (ko00940) or flavonoid biosynthesis (ko00941) with correlation coefficients > 0.8 were performed separately using the CCA package in R software (http://www.r-project.org).

Genes with similar expression patterns are often functionally related. We performed coexpression analysis of genes involved in lignin, cellulose, and hemicellulose biosynthesis and of TFs including NAC, MYB, AUX-IAA, and WRKY to identify TFs involved in the regulation of target gene expression using Cytoscape 3.7.1 (https://cytoscape.org/). Another coexpression analysis was performed on genes involved in monolignol biosynthesis and monolignol transport to identify *PgrABCGs* that could be involved in the transmembrane transport of monolignols.

### Quantitative real-time PCR (qRT-PCR) analysis

The total RNA was extracted from inner and outer seed coats of “Dabenzi” and “Tunisia” pomegranate seeds at 50, 95, and 140 DAF using TRIzol reagent (Invitrogen, Carlsbad, USA). First-strand cDNA was synthesized using the PrimeScript™ RT Reagent Kit (TaKaRa, China) according to the manufacturer’s instructions. TB Green^®^ Premix Ex Taq™ (TaKaRa, China) was used to perform qRT-PCR on an ABI StepOne Plus (Applied Biosystems, Foster, USA). Relative quantification of specific mRNA levels was performed using the cycle threshold (Ct) 2(^−ΔΔCt^) method with the pomegranate actin gene (OWM91407) as an internal control. The primers used for qRT-PCR were designed using Primer Premier 5.0 software (http://www.premierbiosoft.com) and are shown in Supplementary Table S2. Relative expression of candidate genes was illustrated using GraphPad Prism 6.01 (GraphPad Software Inc.).

## Supplementary information

Supplementary Figures

Supplementary Tables

## Data Availability

The RNA-seq data have been deposited in the NCBI Sequence Read Archive under accession number PRJNA548841.
