# Supplementary material for: Diversity of metabolite accumulation patterns in inner and outer seed coats of pomegranate: exploring their relationship with genetic mechanisms of seed coat development
Source: Hortic Res. 2020 Jan 7;7:10. doi: 10.1038/s41438-019-0233-4 (PMC6946660; doi:10.1038/s41438-019-0233-4)
Supplement: Supplementary file 1 — Supplementary Figures [file 41438_2019_233_MOESM1_ESM.docx]

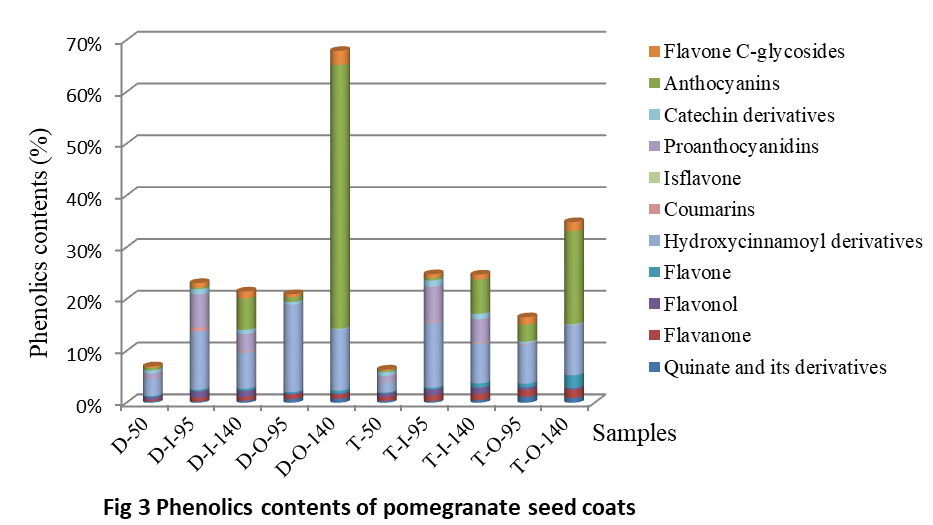


Figure S1 Phenolic compound contents in the inner and outer seed coats at different development stages


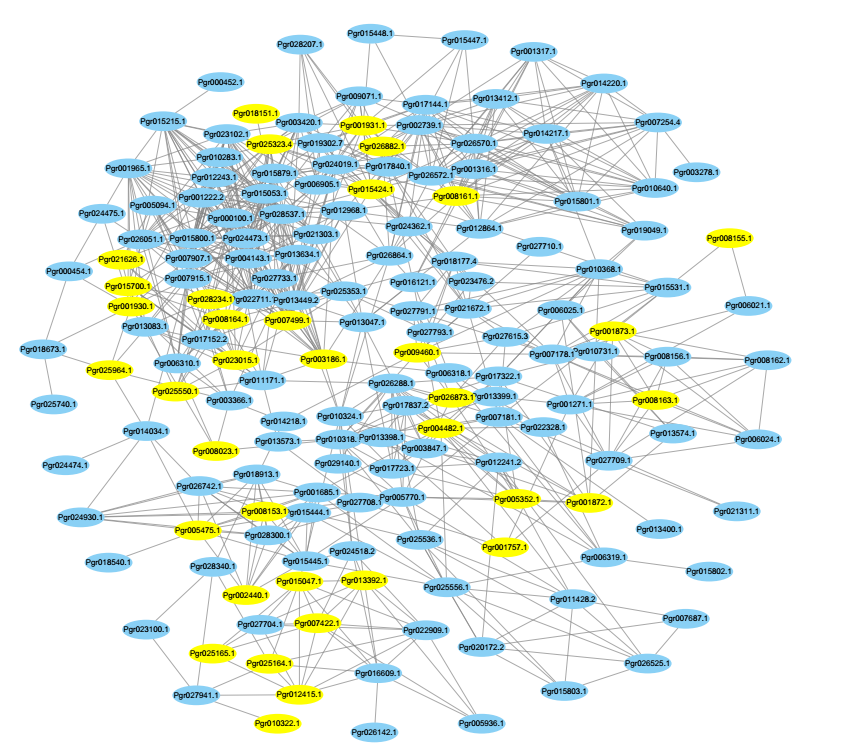


Figure S2 Co-expression of *PgrABCGs* and genes involving in lignin biosynthesis

Genes in yellow ovals were *PgrABCGs,* genes in blue ovals were genes in lignin biosynthesis pathway.


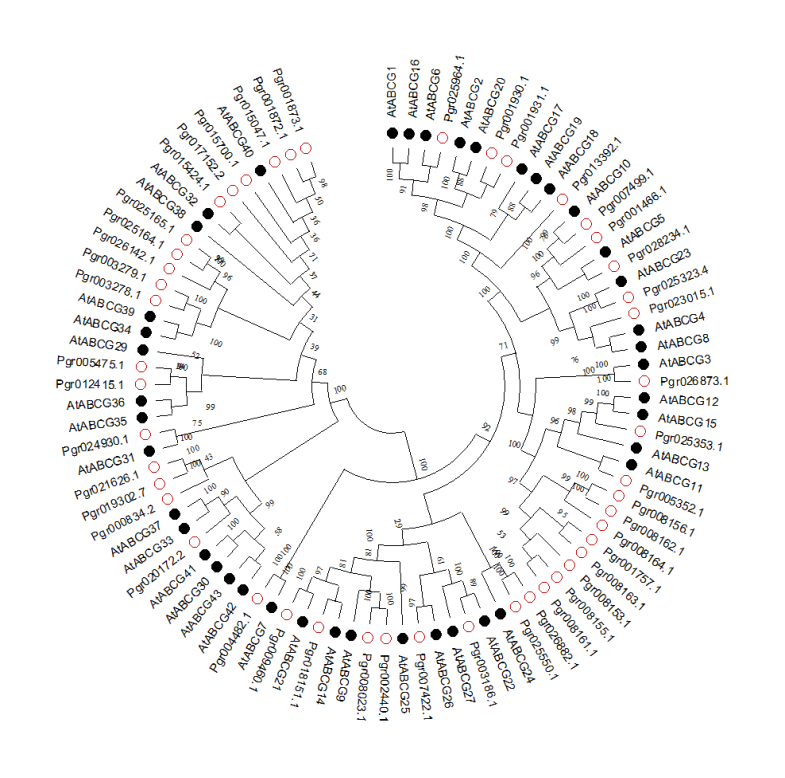


Figure S3 Phylogenetic tree of *PgrABCGs* and *AtABCGs*


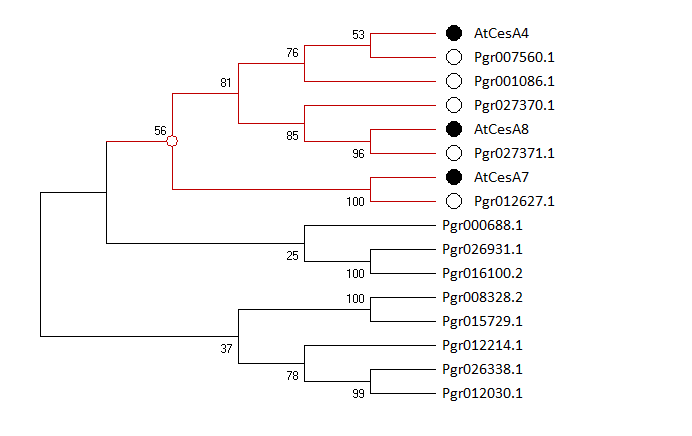


Figure S4 Phylogenetic tree of CesA


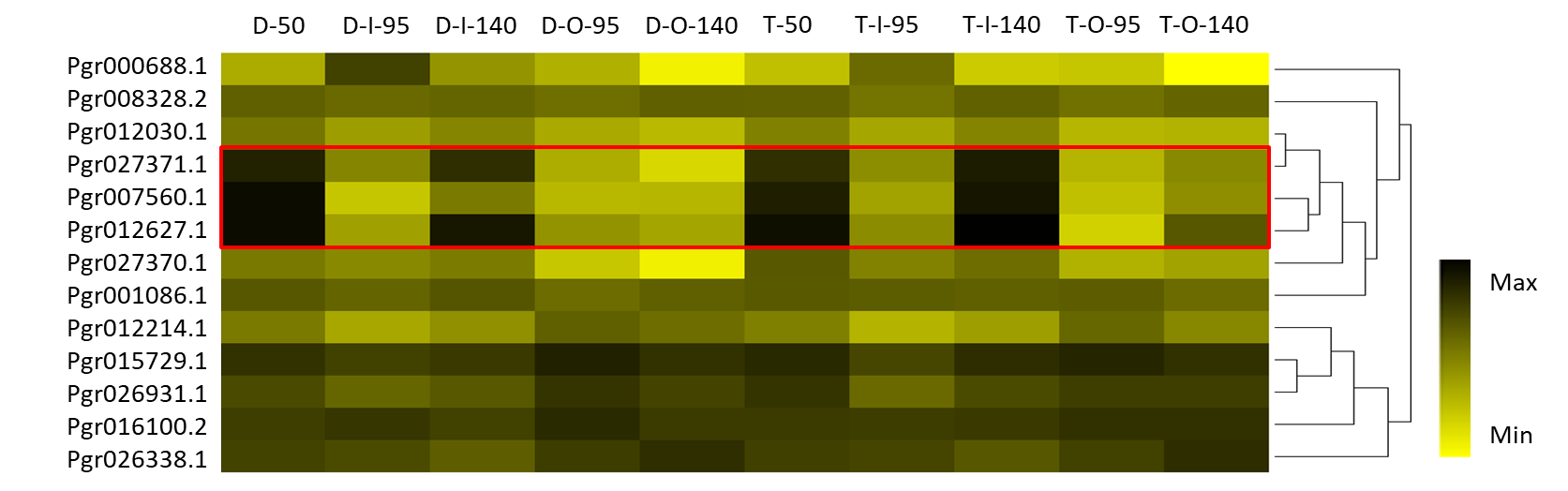


Figure S5 Expression profiles of CesA in inner seed coats and outer seed coats of pomegranate


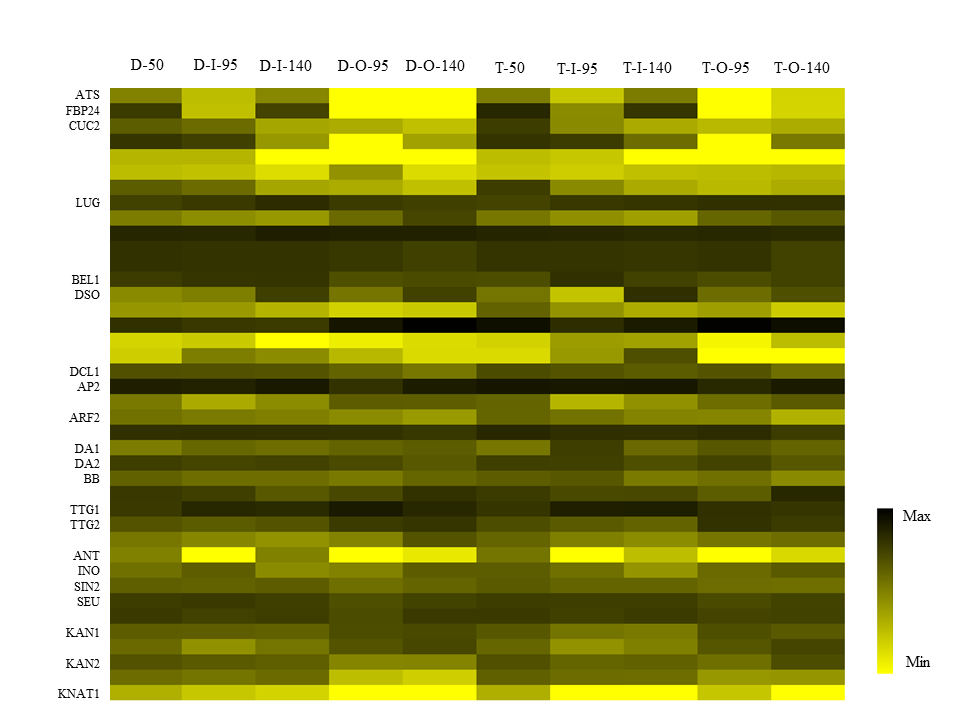


Figure S6 Expression patterns of genes involved in seed coat development. RNAs isolated from inner and outer seed coats at 50, 95, and 140 DAF of ‘Dabenzi’ and ‘Tunisia’ pomegranates were used to survey the expression patterns of genes potentially involved in inner and outer seed coat development. The letters D and T on the x-axis represent the pomegranate cultivars ‘Dabenzi’ and ‘Tunisia’; I and O represent inner and outer seed coat; and 50, 95, and 140 represent the days after flowering. The names of candidate genes are shown on the y-axis. Gene expression is represented as Log_2_FPKM (fragments per kilo base of exon per million fragments mapped).

*
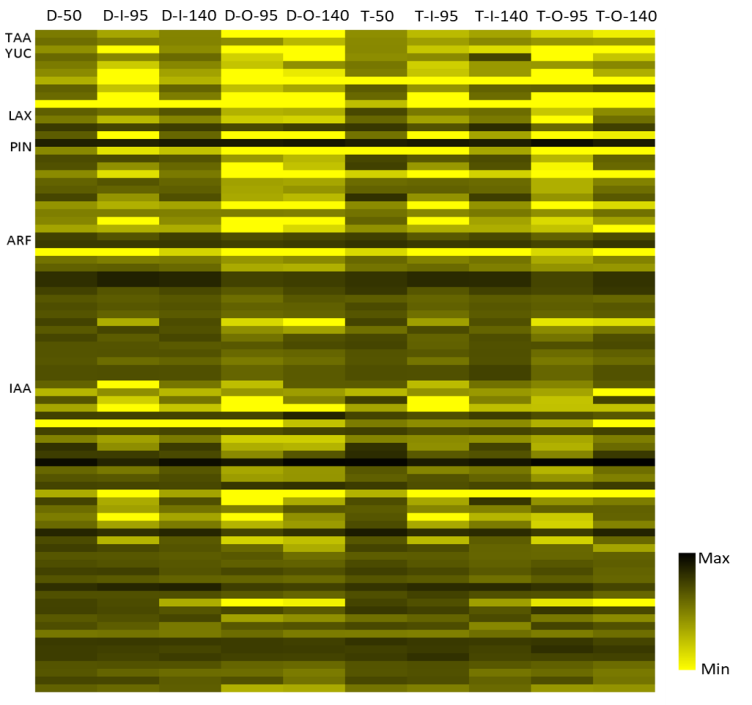
*

Figure S7 Expression profiling of genes involving in auxin biosynthesis, transport and signaling in seed coats during fruit development. RNAs isolated from inner and outer seed coats at 50, 95, and 140 DAP of ‘Dabenzi’ and ‘Tunisi’ respectively, were used to survey the expression patterns of genes involving in auxin biosynthesis, transport and signaling in seed coats during fruit development. The letter D and T in T axis represented cultivars ‘Dabenzi’ and ‘Tunisi’, I and O represent inner seed coat and outer seed coat, 50,95 and 140 represent the DAP of fruits respectively. The Y-axis is the enzymes encoded by candidate genes. Gene expression was represented as Log_2_FPKM (fragments per kilobase of exon per million fragments mapped).
