# Supplementary material for: Diversity of metabolite accumulation patterns in inner and outer seed coats of pomegranate: exploring their relationship with genetic mechanisms of seed coat development
Source: Hortic Res. 2020 Jan 7;7:10. doi: 10.1038/s41438-019-0233-4 (PMC6946660; doi:10.1038/s41438-019-0233-4)
Supplement: Supplementary file 2 — Supplementary Tables [file 41438_2019_233_MOESM2_ESM.docx]

**Table S1** Samples used for transcripts and metabolic profiling

| Cultivars | Tissues | Developmental stages(DAF^†^) | Abbreviation |
| --- | --- | --- | --- |
| *P. granatum* ‘Dabenzi’ | Inner and outer seed coats | 50 | D-50 |
|  | Inner seed coats | 95 | D-I-95 |
|  |  | 140 | D-I-140 |
|  | Outer seed coats | 95 | D-O-95 |
|  |  | 140 | D-O-140 |
| *P. granatum* ‘Tunisia’ | Inner and outer seed coats | 50 | T-50 |
|  | Inner seed coats | 95 | T-I-95 |
|  |  | 140 | T-I-140 |
|  | Outer seed coats | 95 | T-O-95 |
|  |  | 140 | T-O-140 |

† Days after flowering（DAF）.

**Table S2** Primers used in the qRT-PCR

| Gene ID | Primes | Sequences(5’→3’) |
| --- | --- | --- |
| Pgr006319 | Pgr006319-F | GCCACATTGCCGTCAAGTT |
|  | Pgr006319-R | TGGACATAGCGGACTGCATT |
| Pgr022711.1 | Pgr022711-F | ACGGACCACTTGACGCATAA |
|  | Pgr022711-R | TCTCGGCCTCTTTGACGTAA |
| Pgr011127.1 | Pgr011127-F | AAGAACAGCCAAGCAGCGTT |
|  | Pgr011127-R | GTTCGGTTGGTGGAGACTGAA |
| Pgr007776.1 | Pgr007776-F | CTTGCTGCCCGATGAGAAC |
|  | Pgr007776-R | TGCTCCTGTAAATGCCGTTG |
| Pgr010025.1 | Pgr010025-F | GCATGCTATCAAGACCCGAGA |
|  | Pgr010025-R | CTTCCAGTGGCCAATAAGTCC |
| Pgr008155.1 | Pgr008155-F | CATCCGAGAAATGGGCCTAA |
|  | Pgr008155-R | AGCTTCGGGTGGGTCAATAA |
| Pgr001940.1 | Pgr001940-F | AGTGAAAAGGGCGTGTTGG |
|  | Pgr001940-R | TCCCGTCCTGGCATTGTTA |
| Pgr027753.1 | Pgr027753-F | TCGAGATTTGTGGCAAGGATTC |
|  | Pgr027753-R | ACTTTGGCGTCGCTCTTTCA |
| Pgr011171.1 | Pgr011171-F | CCCGGAAGTGTCGTGAGATA |
|  | Pgr011171-R | TGATTTCCGGAGGGGTCTT |
| Pgr006803.1 | Pgr006803-F | GAGAACGGGTTCCTGAGCTTA |
|  | Pgr006803-R | CTCGCGCCCATATATGTGAG |
